# Foliar-Applied LW-CNs Alleviate Cadmium Toxicity in Soybean by Regulating Cd Accumulation, Photosynthesis and Nitrogen Metabolism

**DOI:** 10.3390/plants15213282

**Published:** 2026-10-24

**Authors:** Xiaoyu Zhu, Mengyang Li, Zhiqian Li, Wenya Wang, Linfeng Bao, Uzma Aslam, Hengbin Zhang, Yunlong Zhai

**Affiliations:** 1 College of Biological Science and Technology, Yili Normal University, Yining 835000, China; zhuxiaoyuyyds@163.com; 2 Key Laboratory of Genetic Improvement and Efficient Production for Specialty Crops in Arid Southern Xinjiang of Xinjiang Corps, Alar 843300, China; 18746229985@163.com (M.L.); 18530201985@163.com (Z.L.); uzmaaslam522@gmail.com (U.A.); 3 College of Agriculture, Anhui Agricultural University, Hefei 230036, China; 18317846805@163.com; 4 Key Laboratory of Soybean Biology in Chinese Ministry of Education, College of Agriculture, Northeast Agricultural University, Harbin 150030, China; blfbzb@163.com; 5 Crops Research Institute, Xinjiang Academy of Agricultural and Reclamation Science, Shihezi 832000, China

**Keywords:** soybean, cadmium stress, licorice–wolfberry nanocomposite (LW-CNs), nitrogen metabolism

## Abstract

Cadmium (Cd) pollution seriously threatens food safety. Plant-derived nanomaterials, due to their environmental friendliness, have emerged as a promising approach for crop stress tolerance regulation; however, their mechanisms under Cd stress remain unclear. In this study, licorice–wolfberry-derived nanocomposites (LW-CNs) were synthesized via a one-step hydrothermal reaction following an established protocol, and their physicochemical properties have been characterized in our previous studies. These were foliarly applied at 200 mg/L to soybean seedlings under 300 μmol/L Cd stress, and the physiological mechanisms underlying their alleviation of Cd toxicity were systematically analyzed. The results showed that Cd stress significantly inhibited soybean seedling growth, reducing net photosynthetic rate from 6.42 to 0.29 μmol·m^−2^·s^−1^, and induced substantial accumulation of H_2_O_2_, O_2_·^−^, and MDA in leaves. After LW-CN application, plant height, shoot fresh weight, and root fresh weight increased by 5.8%, 29.0%, and 28.2%, respectively, while Pn recovered to 0.94 μmol·m^−2^·s^−1^. Mechanistic analysis revealed that the ameliorative effects of LW-CNs resulted from four synergistic actions: (i) maintaining chloroplast ultrastructural integrity, with Pn recovery accompanied by stable intercellular CO_2_ concentration, suggesting that the relief of photosynthetic limitation was unlikely to be attributable to stomatal factors alone; (ii) systemically upregulating SOD, POD, CAT, APX, DHAR, and GR activities, modulating the redox status of the AsA-GSH cycle, and significantly reducing H_2_O_2_ (2.77 to 2.14 μmol/g), O_2_·^−^ (284.73 to 247.40 nmol/g), and MDA (79.29 to 58.40 nmol/g) contents, thereby maintaining ROS homeostasis; (iii) decreasing Cd concentrations in roots, stems, and leaves, and reducing chloroplast Cd co-localization signals (0.774 to 0.171), thus mitigating direct damage to photosynthetic machinery at the source; and (iv) organ-specifically reconfiguring nitrogen metabolism, enhancing NR, GOGAT, and GDH activities and glutamate accumulation in roots, and promoting nitrogen redistribution among roots, stems, and leaves. These findings provide a multidimensional physiological framework—covering Cd-accumulation reduction, photosynthesis protection, redox stabilization, and nitrogen redistribution—for understanding how a plant-derived nanomaterial enhances soybean Cd tolerance, and offer a reference for developing plant-based strategies to sustain soybean production in Cd-contaminated farmlands.

## 1. Introduction

By 2050, global food crop production must increase by more than 50% to meet rising human demand—a target contingent upon synergistic improvements across multiple agronomic factors. While fertilizer-responsive crop varieties developed during the Green Revolution have substantially increased grain yields and sustained population growth, anthropogenic land degradation—particularly heavy metal contamination—has intensified markedly. Cadmium (Cd), a non-biodegradable and highly persistent toxic metal primarily derived from mining and industrial activities, now poses an escalating threat to agricultural productivity and food safety [1,2,3]. Contaminated water resources are among the principal pathways through which Cd and other heavy metals enter agricultural environments [4], and the chemical mechanisms of Cd toxicity and detoxification in plants—including chelation, sequestration, and transporter-mediated compartmentalization—have been reviewed in detail [5]. Although numerous foliar-applied formulations have been designed to enhance Cd tolerance in crops, many rely on chemically synthesized precursors and may co-contain trace heavy metals, thereby introducing substantial risks of secondary contamination. Consequently, developing eco-compatible, biologically derived strategies to improve crop Cd tolerance is not only scientifically urgent but also essential for long-term food security [6,7,8].

Studies on engineered nanomaterials for mitigating heavy metal stress in crops have expanded rapidly [9,10,11,12], and nanomaterial-based approaches for the remediation of heavy-metal-polluted soils have advanced in parallel [13]. For example, rhizospheric application of 100 μg/mL Mn_3_O_4_ nanoparticles significantly alleviates growth inhibition in Arabidopsis thaliana under Cd stress. Foliar application of Mn_3_O_4_ nanoparticles enhances photosynthetic efficiency—including maximum quantum yield of PSII and net CO_2_ assimilation rate—in rice exposed to Cd [14]. Zinc oxide (ZnO) nanoparticles improve Cd tolerance in lettuce by modulating carbon (C), nitrogen (N), and phosphorus (P) metabolism in leaves and roots, while concurrently enriching beneficial endophytes and rhizosphere microbiota [15]. Moreover, Hu et al. demonstrated that co-application of foliar ZnO nanoparticles and plant growth-promoting rhizobacteria synergistically enhances Cd tolerance in cotton, outperforming either treatment alone [16]. However, conventional nanomaterial synthesis often involves energy-intensive processes and hazardous reagents, raising concerns about environmental footprint and scalability. To address this, Shang et al. developed a Se-enriched nanocomposite using Aloe vera extract as a green reducing and capping agent, which enhanced Cd tolerance in rice while minimizing reliance on synthetic chemicals [7]. Nevertheless, whether fully plant-derived nanomaterials can consistently confer robust, mechanism-based Cd tolerance across diverse crop species remains unresolved—and the molecular and physiological bases of their action require systematic investigation [6,17].

Cd stress impairs crop growth through dual constraints: the suppression of biomass accumulation and the inhibition of photosynthetic capacity [18,19]. At the cellular level, Cd-induced overproduction of reactive oxygen species (ROS) disrupts redox homeostasis, damages membranes and proteins, and triggers oxidative signaling cascades that impair cell division and expansion. Thus, ROS scavenging and redox regulation constitute central mechanisms underlying nanomaterial-mediated Cd tolerance [9,20,21]. Concurrently, limiting Cd influx into root cells and restricting its translocation to shoots represent critical physiological strategies for detoxification [22,23,24]. X. Zhang et al. reported that exogenous application of zinc oxide nanoparticles (ZnO NPs) reduced root-to-shoot Cd translocation and increased shoot biomass under Cd stress. Furthermore, Cd severely compromises N acquisition and assimilation—by downregulating nitrate transporters (e.g., NRT1.1, NRT2.1) and key enzymes (e.g., nitrate reductase, glutamine synthetase)—thereby limiting amino acid synthesis, chlorophyll biosynthesis, and ultimately photosynthetic performance [3,25,26]. Root-applied nanomaterials were shown to restore the expression of N transporter genes and enhance N uptake efficiency in Cd-stressed plants, supporting sustained growth. Licorice–wolfberry-based nanocomposites (LW-CNs) were first identified as potent elicitors of jasmonic acid (JA)-mediated defense responses, conferring resistance to Sclerotinia sclerotiorum in rapeseed (Brassica napus). Subsequent studies revealed that LW-CNs enhance thermotolerance in soybean (Glycine max) by stabilizing photosystem II and upregulating heat shock proteins (HSPs), and improve salt tolerance via the modulation of Na^+^/K^+^ homeostasis and antioxidant enzyme activity (e.g., SOD, CAT, APX). Critically, these effects are consistently associated with improved ROS homeostasis—suggesting a conserved, stress-agnostic mode of action. Given their demonstrated efficacy across biotic and abiotic stresses, and their capacity to fine-tune redox balance, we hypothesize that LW-CNs will similarly mitigate Cd toxicity in soybean by coordinately regulating ROS metabolism, Cd subcellular compartmentalization, and N assimilation pathways.

Soybean is a globally indispensable oilseed and leguminous protein source, supplying approximately 30% of the world’s plant-derived protein and serving as a major dietary protein source for more than two billion people [19,27,28]. Enhancing its resilience to Cd contamination is therefore pivotal—not only for maintaining yield stability in contaminated agroecosystems but also for safeguarding nutritional quality and minimizing dietary Cd exposure [29,30]. In this study, we applied licorice–wolfberry-based nanocomposites to Cd-stressed soybean seedlings via standardized foliar spraying, with three specific aims: (1) to quantify the growth-promoting and growth-protective effects of LW-CNs under Cd stress; (2) to dissect the physiological and biochemical mechanisms underlying LW-CNs-mediated Cd detoxification—including ROS scavenging, activation of the antioxidant system, and reduction in tissue Cd accumulation; and (3) to elucidate how LW-CNs modulate N uptake, assimilation, and reallocation under Cd stress, with emphasis on the activities of nitrogen-metabolizing enzymes and organ-specific nitrogen redistribution. Collectively, this work provides a physiological framework for plant-derived nanomaterials in heavy metal stress mitigation, offering a reference for the development of environmentally sound crop-protection strategies within the broader context of sustainable agriculture [31].

## 2. Results and Analysis

### 2.1. LW-CNs Alleviate Cadmium-Induced Growth Inhibition in Soybean

**Figure 1:**
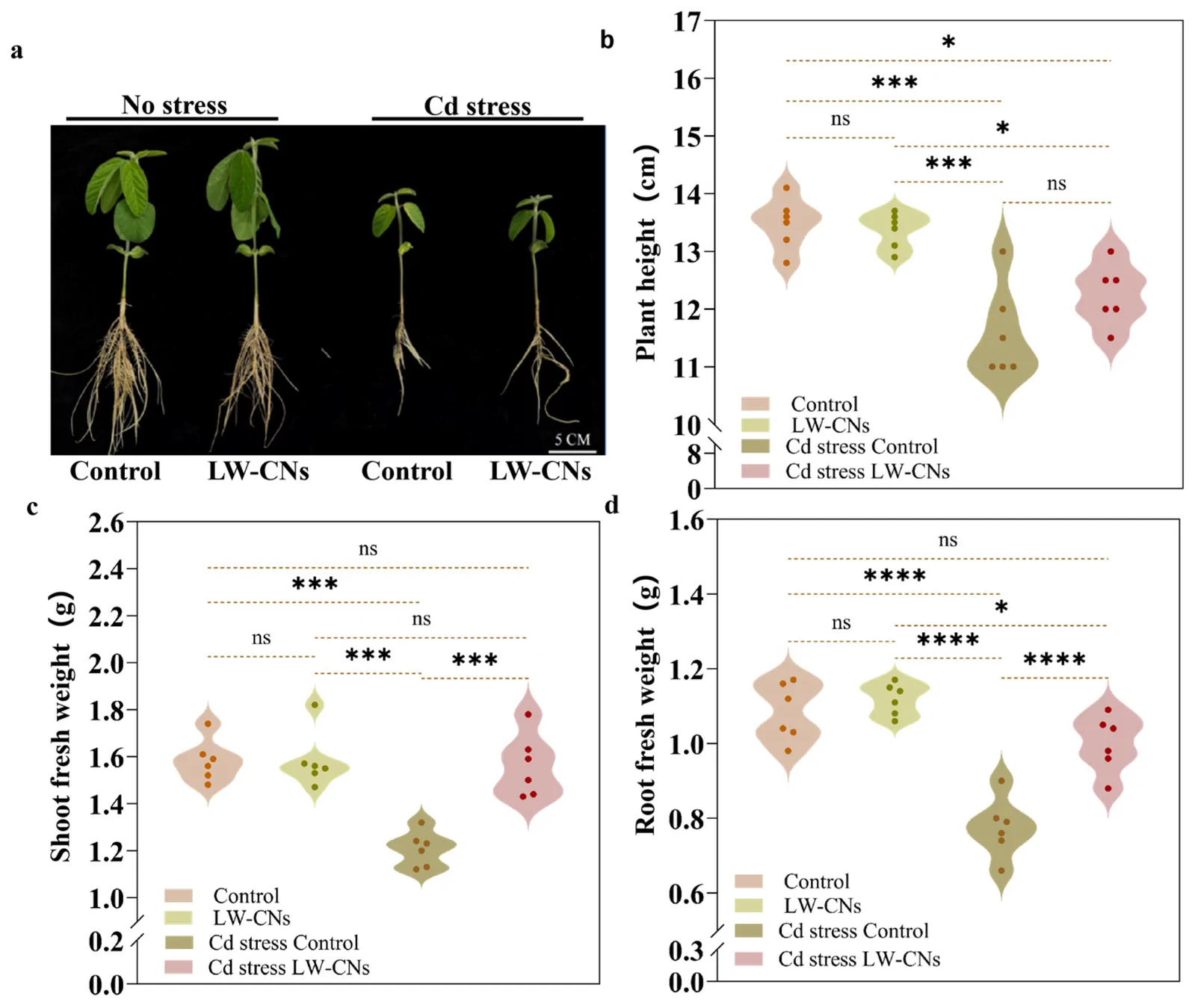
Effects of foliar application of LW-CNs on growth parameters of soybean seedlings under cadmium stress. (**a**) Phenotypic photographs of soybean seedlings under non-stress and cadmium stress conditions; scale bar = 5 cm. (**b**) Plant height. (**c**) Aboveground fresh weight. (**d**) Root fresh weight. Treatment descriptions: control: no cadmium stress, without LW-CNs application; LW-CNs: LW-CNs applied alone (no cadmium stress); Cd stress control: cadmium stress, without LW-CNs application; Cd stress + LW-CNs: cadmium stress with LW-CNs application. Values are means ± SD of six biological replicates. ns, *p* ≥ 0.05; *, *p* < 0.05; **, *p* < 0.01; ***, *p* < 0.001; ****, *p* < 0.0001.

Cadmium stress markedly inhibited the growth of soybean seedlings. The measurements of growth parameters (Figure 1) showed that under the condition without LW-CNs application, Cd treatment significantly reduced the plant height, shoot fresh weight and root fresh weight of soybean seedlings compared with the non-stressed control. Under non-Cd Cd-stress conditions, the plant height (13.37 ± 0.14 cm), shoot fresh weight (1.58 ± 0.05 g), and root fresh weight (1.12 ± 0.02 g) of soybean seedlings after LW-CNs foliar application exhibited no significant differences relative to the blank control group (plant height: 13.48 ± 0.20 cm; shoot fresh weight: 1.58 ± 0.04 g; root fresh weight: 1.08 ± 0.03 g) (Figure 1a–d). Under Cd stress, foliar spraying of LW-CNs significantly alleviated Cd toxicity-induced growth inhibition. For the Cd-stressed control group (without LW-CNs application), the plant height, shoot fresh weight and root fresh weight of soybean seedlings were 11.58 ± 0.36 cm, 1.21 ± 0.03 g and 0.78 ± 0.04 g, respectively. Under Cd stress, LW-CNs treatment significantly increased the plant height (12.25 ± 0.23 cm), shoot fresh weight (1.56 ± 0.06 g), and root fresh weight (1.00 ± 0.03 g) of soybean seedlings (Figure 1a–d).

### 2.2. LW-CNs Maintain Leaf Photosynthetic Gas Exchange Under Cadmium Stress

**Figure 2:**
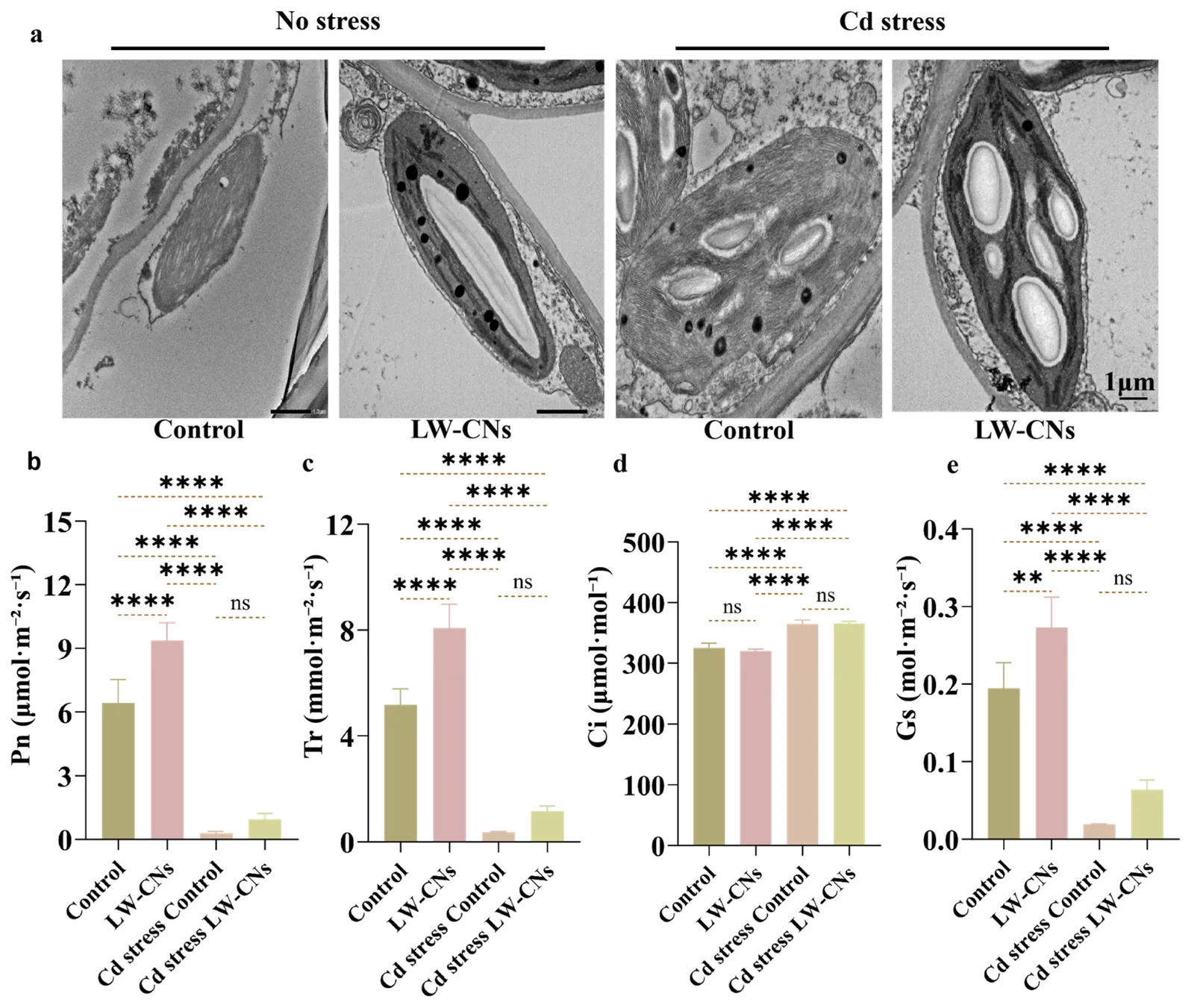
Effects of LW-CNs on chloroplast ultrastructure and photosynthetic parameters in soybean seedlings under cadmium stress. (**a**) Transmission electron micrographs of leaf chloroplasts; scale bar = 1 μm. (**b**) Net photosynthetic rate (Pn), (**c**) transpiration rate (Tr), (**d**) intercellular CO_2_ concentration (Ci), (**e**) stomatal conductance (Gs). Treatment description: control (control group): no cadmium stress, no application of LW-CNs; LW-CNs: sole application of LW-CNs (without cadmium stress); Cd stress control (cadmium stress control group): cadmium stress, no application of LW-CNs; Cd stress + LW-CNs: cadmium stress with application of LW-CNs. Values are means ± SD of three biological replicates. ns, *p* ≥ 0.05; *, *p* < 0.05; **, *p* < 0.01; ***, *p* < 0.001; ****, *p* < 0.0001.

Transmission electron microscopy results revealed that in the absence of LW-CNs application, Cd stress induced severe damage to the chloroplast structure of soybean leaves, which was characterized by disordered thylakoid lamellae, deformed chloroplast morphology and increased abundance of plastoglobuli (Figure 2a). Foliar application of LW-CNs largely preserved the structural integrity of chloroplasts and alleviated Cd-induced phytotoxicity to chloroplasts.

Gas exchange parameter measurements (Figure 2b–e) showed that without LW-CNs application, Cd treatment significantly reduced the net photosynthetic rate, transpiration rate and stomatal conductance of soybean leaves compared with the non-stressed control. The net photosynthetic rate decreased significantly from 6.42 ± 0.55 μmol·m^−2^·s^−1^ to 0.29 ± 0.04 μmol·m^−2^·s^−1^; the transpiration rate declined from 0.0052 ± 0.0003 mol·m^−2^·s^−1^ to 0.00037 ± 0.00001 mol·m^−2^·s^−1^; and stomatal conductance dropped from 0.195 ± 0.016 mol·m^−2^·s^−1^ to 0.020 ± 0.004 mol·m^−2^·s^−1^. Meanwhile, the intercellular CO_2_ concentration increased markedly from 325.30 ± 3.82 μmol·mol^−1^ to 364.50 ± 3.46 μmol·mol^−1^. Under non-Cd Cd-stress conditions, LW-CNs application significantly increased net photosynthetic rate, transpiration rate and stomatal conductance to 9.37 ± 0.42 μmol·m^−2^·s^−1^, 0.0081 ± 0.0005 mol·m^−2^·s^−1^ and 0.273 ± 0.019 mol·m^−2^·s^−1^, respectively, while intercellular CO_2_ concentration showed no significant change (319.93 ± 1.53 μmol·mol^−1^). Under Cd stress, foliar spraying of LW-CNs significantly restored the photosynthetic capacity of soybean seedlings. For the Cd-stressed control group (without LW-CNs application), the net photosynthetic rate, transpiration rate and stomatal conductance were 0.29 ± 0.04 μmol·m^−2^·s^−1^, 0.00037 ± 0.00001 mol·m^−2^·s^−1^ and 0.020 ± 0.004 mol·m^−2^·s^−1^, respectively. Under Cd stress, LW-CNs treatment markedly elevated these parameters to 0.94 ± 0.14 μmol·m^−2^·s^−1^, 0.0011 ± 0.0001 mol·m^−2^·s^−1^ and 0.064 ± 0.006 mol·m^−2^·s^−1^. Nevertheless, no significant difference was observed in intercellular CO_2_ concentration between the two Cd-stressed groups (364.50 ± 3.46 μmol·mol^−1^ vs. 364.89 ± 2.00 μmol·mol^−1^).

### 2.3. LW-CNs Maintained ROS Homeostasis in Soybean Under Cadmium Stress

**Figure 3:**
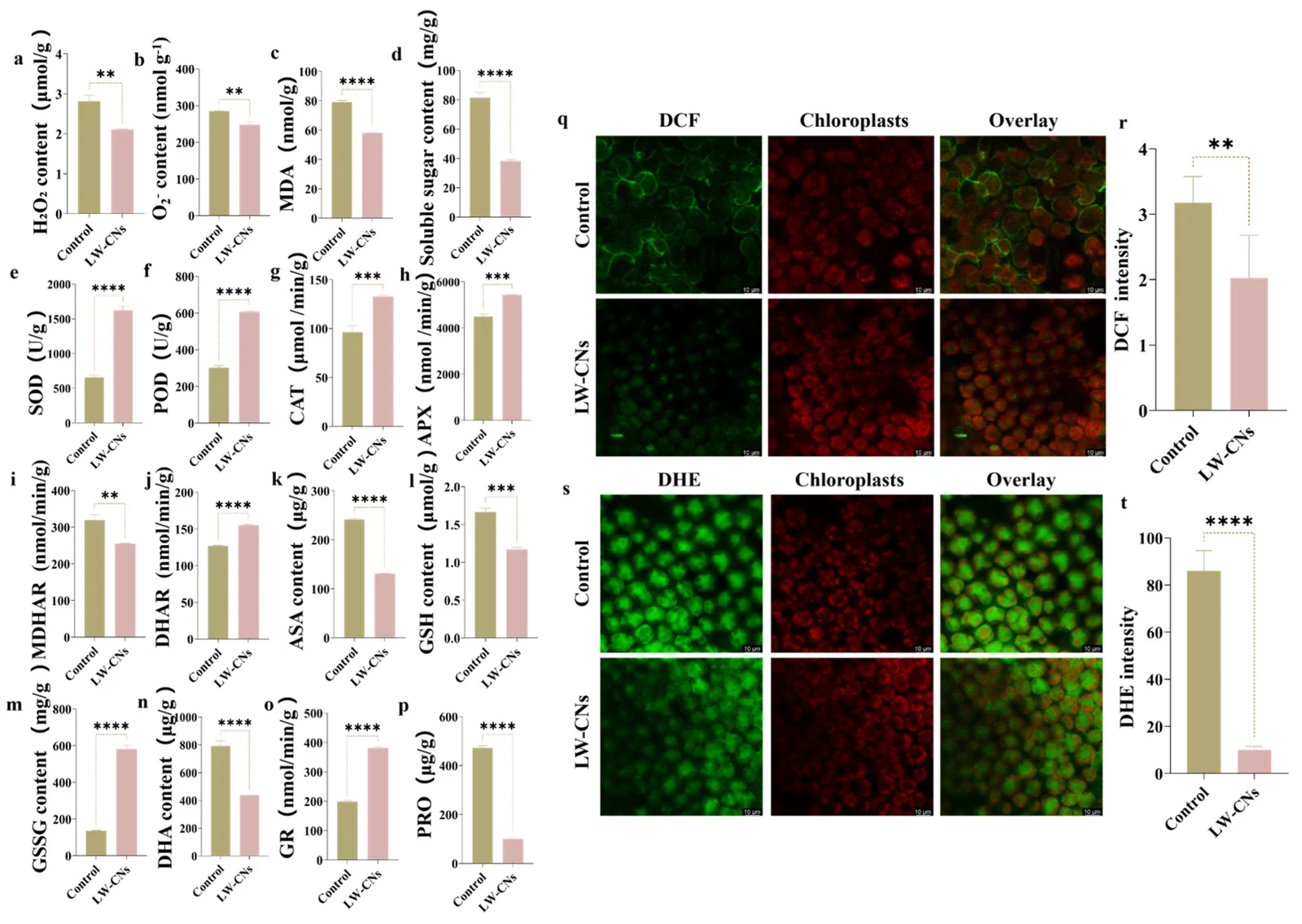
The effects of LW-CNs on oxidative damage, antioxidant enzyme activities, non-enzymatic antioxidant contents, reactive oxygen species (ROS) fluorescence imaging, and fluorescence intensity in the leaves of soybean seedlings under cadmium stress. (**a**) Hydrogen peroxide (H_2_O_2_) content; (**b**) superoxide anion (O_2_·^−^) content; (**c**) malondialdehyde (MDA) content; (**d**) soluble Soluble sugar content; (**e**) superoxide dismutase (SOD) activity; (**f**) peroxidase (POD) activity; (**g**) catalase (CAT) activity; (**h**) ascorbate peroxidase (APX) activity; (**i**) monodehydroascorbate reductase (MDHAR) activity; (**j**) dehydroascorbate reductase (DHAR) activity; (**k**) reduced ascorbic acid (ASA) content; (**l**) reduced glutathione (GSH) content; (**m**) oxidized glutathione (GSSG) content; (**n**) dehydroascorbic acid (DHA) content; (**o**) glutathione reductase (GR) activity; (**p**) proline (PRO) content. (**q**,**r**) In situ visualization and quantitative analysis of H_2_O_2_ accumulation using DCF fluorescent probe. (**q**) Representative confocal images: DCF fluorescence (green, H_2_O_2_ signal), chloroplast autofluorescence (red), and merged overlay channels in control and LW-CNs-treated leaves. (**r**) Quantification of relative DCF fluorescence intensity. (**s**,**t**) In situ visualization and quantitative analysis of O_2_·^−^ accumulation using DHE fluorescent probe. (**s**) Representative confocal images: DHE fluorescence (green, O_2_·^−^ signal), chloroplast autofluorescence (red), and merged overlay channels in control and LW-CNs-treated leaves. (**t**) Quantification of relative DHE fluorescence intensity. Scale bar = 10 μm. The bar graphs on the right represent DCF fluorescence intensity and DHE fluorescence intensity, respectively. Treatment description: control: cadmium-stressed plants without LW-CN foliar application; LW-CNs: cadmium-stressed plants with foliar spraying of LW-CNs. Values are means ± SD of three biological replicates. ns, *p* ≥ 0.05; *, *p* < 0.05; **, *p* < 0.01; ***, *p* < 0.001; ****, *p* < 0.0001.

Under cadmium stress, exogenous foliar application of LW-CNs markedly reduced the accumulation of hydrogen peroxide (H_2_O_2_), superoxide anion (O_2_·^−^), malondialdehyde (MDA), and soluble sugars in soybean seedling leaves. Relative to the Cd-stressed control, the H_2_O_2_ concentration decreased from 2.77 ± 0.12 μmol g^−1^ to 2.14 ± 0.09 μmol g^−1^; the O_2_·^−^ level declined from 284.73 ± 1.24 nmol g^−1^ to 247.40 ± 4.80 nmol g^−1^; MDA content dropped from 79.29 ± 0.70 nmol g^−1^ to 58.40 ± 0.28 nmol g^−1^; and soluble sugar content was reduced from 81.10 ± 1.83 mg g^−1^ to 38.10 ± 0.18 mg g^−1^ (Figure 3a–d). The activities of superoxide dismutase (SOD), peroxidase (POD), catalase (CAT), ascorbate peroxidase (APX), dehydroascorbate reductase (DHAR), and glutathione reductase (GR) in the LW-CNs-treated group reached 1622.72 ± 2.65 U g^−1^, 607.71 ± 0.52 U g^−1^, 132.20 ± 0.14 μmol min^−1^ g^−1^, 5436.05 ± 5.20 nmol min^−1^ g^−1^, 155.36 ± 0.12 nmol min^−1^ g^−1^, and 381.79 ± 1.05 nmol min^−1^ g^−1^, respectively, all of which were significantly higher than those in the Cd-stressed control (SOD: 655.15 ± 14.72 U g^−1^; POD: 302.38 ± 4.57 U g^−1^; CAT: 96.34 ± 3.72 μmol min^−1^ g^−1^; APX: 4494.97 ± 59.04 nmol min^−1^ g^−1^; DHAR: 126.79 ± 0.50 nmol min^−1^ g^−1^; GR: 199.54 ± 1.71 nmol min^−1^ g^−1^) (Figure 3e–h,j,o). The LW-CNs treatment lowered the levels of monodehydroascorbate reductase (MDHAR), ascorbic acid (ASA), reduced glutathione (GSH), dehydroascorbic acid (DHA), and proline (PRO). Specifically, MDHAR activity decreased from 319.20 ± 17.42 nmol min^−1^ g^−1^ to 254.75 ± 0.34 nmol min^−1^ g^−1^; ASA content declined from 241.41 ± 2.31 μg g^−1^ to 130.55 ± 1.63 μg g^−1^; GSH content fell from 1.67 ± 0.05 μmol g^−1^ to 1.17 ± 0.02 μmol g^−1^; DHA content dropped from 791.42 ± 26.81 μg g^−1^ to 437.19 ± 0.47 μg g^−1^; and PRO content was reduced from 472.73 ± 7.32 μg g^−1^ to 100.14 ± 2.34 μg g^−1^. In contrast, oxidized glutathione (GSSG) content increased markedly from 135.13 ± 1.09 mg g^−1^ to 580.94 ± 0.77 mg g^−1^ (Figure 3i,k–n).

Under cadmium stress, strong DCF and DHE fluorescence signals were observed in leaf cells of control plants (Figure 3q,s). Quantitative fluorescence analysis revealed that the DCF fluorescence intensity of the cadmium-stressed control was 3.18 ± 0.18, and the DHE fluorescence intensity was 85.24 ± 3.60. After foliar spraying with LW-CNs, the green fluorescence intensities of DCF and DHE in the leaves were substantially attenuated; the DCF fluorescence intensity decreased to 2.02 ± 0.29, and the DHE fluorescence intensity was markedly reduced to 10.00 ± 0.73 (Figure 3r,t).

### 2.4. LW-CNs Reduce Cadmium Accumulation in Plant Tissues

**Figure 4:**
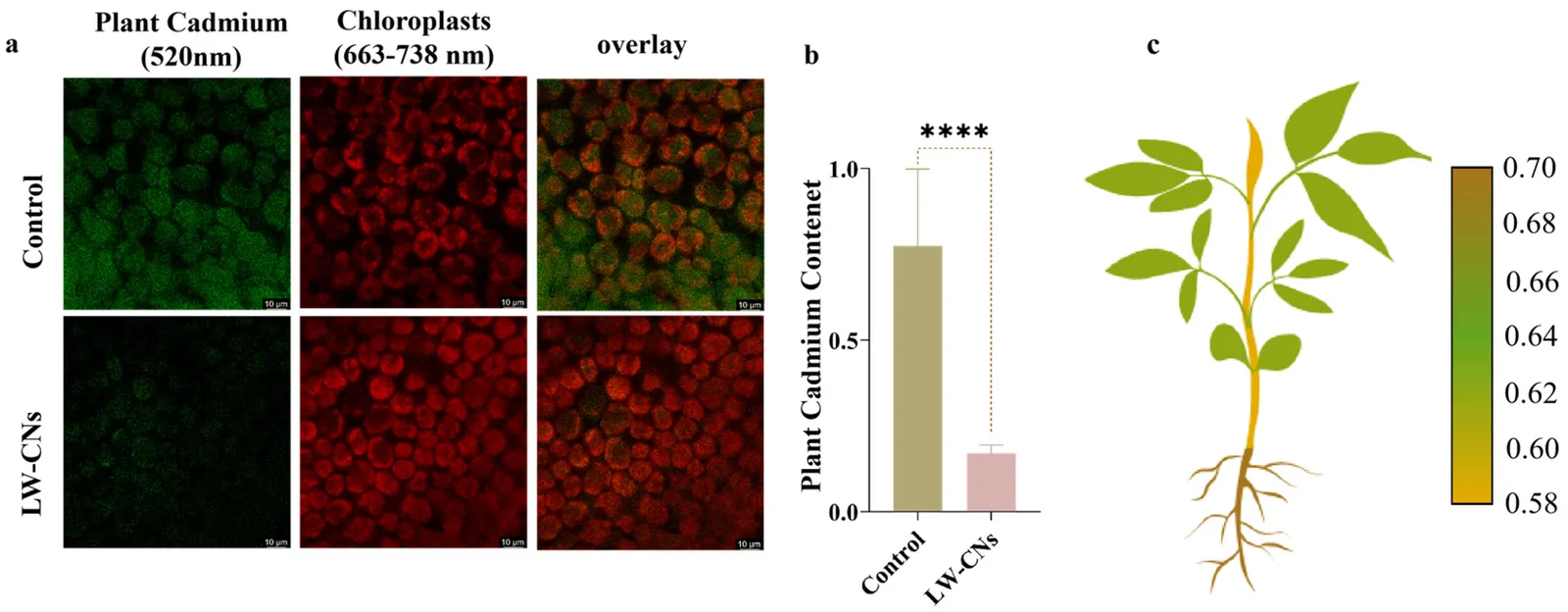
The effects of LW-CNs on cadmium ion fluorescence localization, cadmium accumulation, and cadmium-distribution visualization in soybean leaves under cadmium stress. (**a**) Fluorescence imaging of cadmium ions in leaves. Plant Cadmium (520 nm): the green fluorescence signal of cadmium ions; Chloroplasts (663–738 nm): the red autofluorescence of chloroplasts; Overlay: the merged fluorescence signals. Scale bar = 10 μm. (**b**) A quantitative analysis of the cadmium contents in the soybean plants. (**c**) The cadmium contents in the roots, stems and leaves determined by ICP-MS and expressed on a dry-weight basis (mg·g^−1^ DW). Treatment details: control: cadmium-stressed plants without foliar application of LW-CNs; LW-CNs: cadmium-stressed plants with foliar spraying of LW-CNs. Values are means ± SD of three biological replicates. ns, *p* ≥ 0.05; *, *p* < 0.05; **, *p* < 0.01; ***, *p* < 0.001; ****, *p* < 0.0001.

Distinct green fluorescence signals of cadmium were detected in leaf cells of the cadmium-stressed control group, which exhibited partial co-localization with chloroplasts. The relative fluorescence intensity of cadmium co-localized within chloroplasts was 0.774 ± 0.080 (Figure 4a). Upon foliar application of LW-CNs, the intensity of cadmium-derived green fluorescence in the leaves decreased significantly, and the co-localized fluorescence intensity between cadmium and chloroplasts dropped to 0.171 ± 0.009 (Figure 4b). Quantitative determination of plant cadmium contents was consistent with the fluorescence observations. ICP-MS quantification showed that cadmium concentrations in the roots, stems, and leaves of the Cd-stressed control were 0.697 ± 0.001, 0.583 ± 0.001, and 0.642 ± 0.011 mg·g^−1^ dry weight, respectively, and LW-CN treatment markedly reduced cadmium accumulation in these tissues (Figure 4c). It should be noted that the Leadmium™ Green AM probe provides semi-quantitative information on subcellular Cd distribution; the fluorescence data were therefore interpreted qualitatively and in combination with ICP-MS measurements of total tissue Cd.

### 2.5. LW-CNs Regulate Nitrogen Absorption, Translocation and Assimilation Metabolism in Soybean Seedlings Under Cadmium Stress

**Figure 5:**
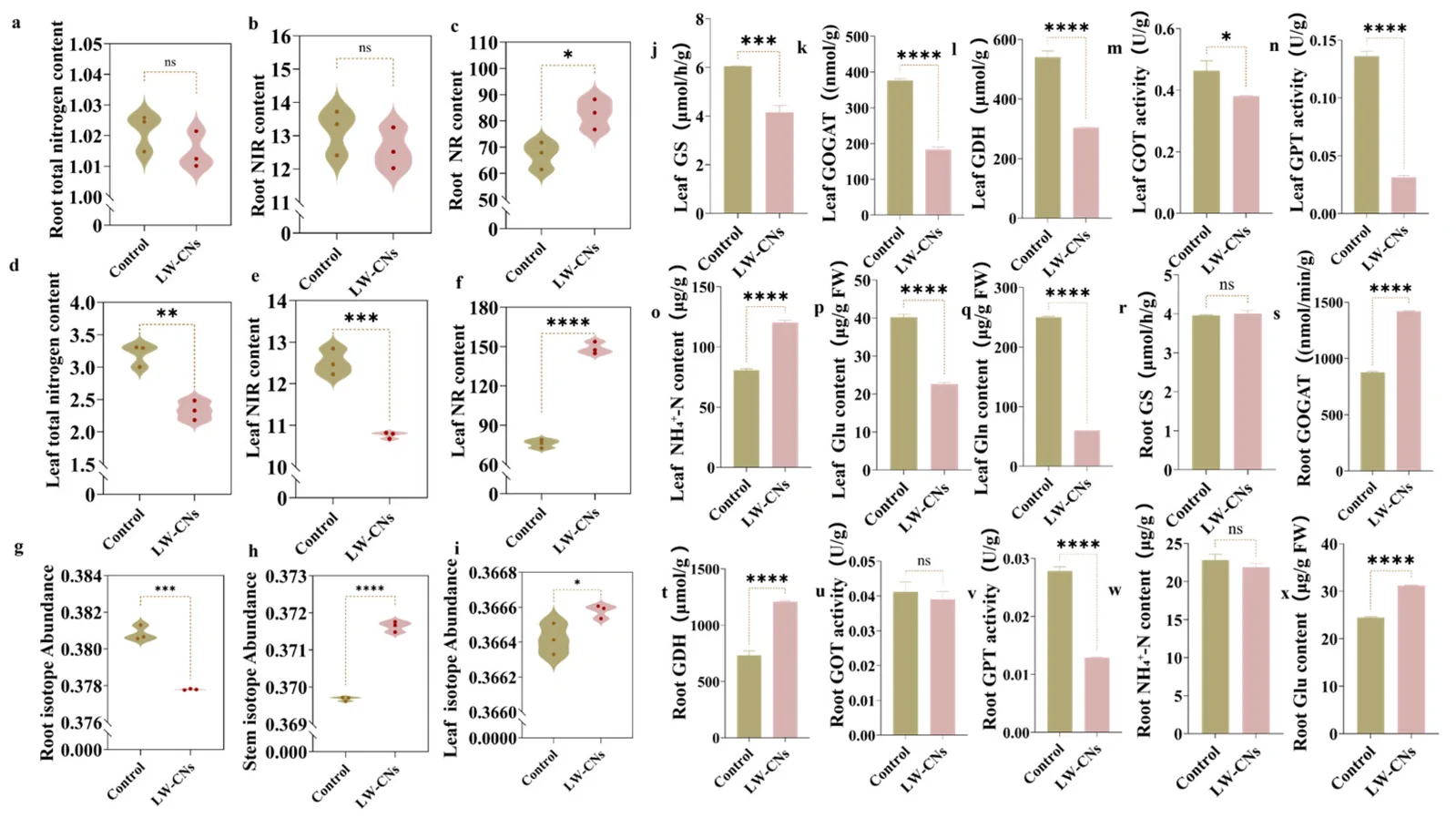
Effects of LW-CNs on nitrogen content, nitrogen-metabolizing enzyme activities, and nitrogen-isotope abundance in soybean seedlings under cadmium stress. (**a**–**c**) Root total nitrogen content, root nitrite reductase (NiR) activity, and root nitrate reductase (NR) activity; (**d**–**f**) leaf total nitrogen content, leaf nitrite reductase (NiR) activity, and leaf nitrate reductase (NR) activity; (**g**–**i**) nitrogen-isotope abundance in roots, stems and leaves; (**j**–**x**) activities of nitrogen-metabolism-related enzymes and contents of nitrogen metabolites in leaves and roots. GS: glutamine synthetase; GOGAT: glutamate synthase; GDH: glutamate dehydrogenase; GOT: glutamic-oxaloacetic transaminase; GPT: glutamic-pyruvic transaminase; NH_4_^+^-N: ammonium nitrogen; Glu: glutamate; Gln: glutamine. Treatment details: control: cadmium-stressed plants without foliar application of LW-CNs; LW-CNs: cadmium-stressed plants with foliar spraying of LW-CNs. Values are means ± SD of three biological replicates. ns, *p* ≥ 0.05; *, *p* < 0.05; **, *p* < 0.01; ***, *p* < 0.001; ****, *p* < 0.0001.

To explore the effects of LW-CNs on nitrogen uptake, translocation and partitioning in soybean seedlings, a set of physiological indices related to nitrogen metabolism were determined. The results showed that the total nitrogen content in roots of cadmium-stressed control plants was 1.02 ± 0.00, compared with 1.01 ± 0.01 in the LW-CNs-treated group. The root nitrite reductase (NiR) activity was 13.17 ± 0.33 in the control and 12.61 ± 0.32 in the LW-CNs group. No significant differences were observed between the two groups for root total nitrogen content and root NiR activity (Figure 5a,b). LW-CNs treatment significantly elevated root nitrate reductase (NR) activity, which increased from 67.03 ± 2.99 in the control to 82.67 ± 3.33 (Figure 5c). In leaves, LW-CNs markedly reduced leaf total nitrogen content (control: 3.20 ± 0.09; LW-CNs: 2.33 ± 0.09) and leaf NiR activity (control: 12.51 ± 0.18; LW-CNs: 10.77 ± 0.05), whereas leaf NR activity increased extremely significantly from 75.9 ± 1.85 in the control to 148.68 ± 2.65 (Figure 5d–f).

Nitrogen stable-isotope abundance results indicated that LW-CNs treatment reduced nitrogen-isotope abundance in roots (control: 0.380 ± 0.001; LW-CNs: 0.378 ± 0.001) (Figure 5g), but significantly enhanced nitrogen-isotope abundance in stems and leaves. Stem nitrogen-isotope abundance rose from 0.370 ± 0.001 (control) to 0.372 ± 0.001 (LW-CNs treatment), and leaf nitrogen-isotope abundance increased from 0.366 ± 0.001 (control) to 0.367 ± 0.001 (LW-CNs treatment) (Figure 5h,i).

In leaves, LW-CNs application significantly decreased the activities of glutamine synthetase (GS), glutamate synthase (GOGAT), glutamate dehydrogenase (GDH), glutamic-oxaloacetic transaminase (GOT), and glutamic-pyruvic transaminase (GPT). Leaf GS activity declined from 6.03 ± 0.01 (control) to 4.15 ± 0.17; GOGAT activity decreased from 372.84 ± 2.17 to 183.20 ± 4.27; GDH activity dropped from 541.17 ± 11.70 to 303.01 ± 1.72; GOT activity fell from 0.46 ± 0.02 to 0.38 ± 0.00; and GPT activity decreased from 0.14 ± 0.00 to 0.03 ± 0.00 (Figure 5j–n). Correspondingly, leaf glutamate (Glu) and glutamine (Gln) contents were significantly lowered (control: 40.15 ± 0.14; LW-CNs: 22.82 ± 0.27) (Figure 5p,q), while ammonium nitrogen (NH_4_^+^-N) content increased remarkably from 80.68 ± 0.37 in the control to 121.42 ± 1.14 (Figure 5o).

Root nitrogen-metabolism indices exhibited an opposite trend relative to leaves. No significant differences were detected between the control and LW-CNs treatments for root GS activity (control: 3.97 ± 0.01; LW-CNs: 4.03 ± 0.03), root GOT activity (control: 0.04 ± 0.00; LW-CNs: 0.04 ± 0.00), and root ammonium nitrogen content (control: 2.29 ± 0.05; LW-CNs: 2.21 ± 0.09) (Figure 5r,u,w). LW-CNs markedly increased root GOGAT and GDH activities: GOGAT activity increased from 878.50 ± 5.80 (control) to 1418.80 ± 7.51, and GDH activity rose from 71.72 ± 1.30 to 120.81 ± 0.38. Meanwhile, LW-CNs inhibited root GPT activity (control: 0.03 ± 0.00; LW-CNs: 0.01 ± 0.00), and root glutamate content increased significantly (control: 24.47 ± 0.10; LW-CNs: 31.22 ± 0.04) (Figure 5s,t,v–x).

## 3. Discussion

### 3.1. LW-CNs Alleviate Cd-Induced Inhibition of Soybean Growth

Cadmium (Cd) is one of the most toxic and mobile heavy metal pollutants in agroecosystems, significantly reducing crop biomass by inhibiting cell division and elongation, disrupting nutrient uptake, and impairing photosynthetic carbon assimilation. In this study, 300 μmol/L Cd stress significantly suppressed soybean seedling height, shoot fresh weight, and root fresh weight, consistent with previous reports across various crops [10,15,32,33]. Foliar application of 200 mg/L LW-CNs restored plant height, shoot fresh weight, and root fresh weight to 105.8%, 129.0%, and 128.2% of the Cd-stressed control, respectively, demonstrating that LW-CNs effectively alleviated Cd-induced growth inhibition. The Cd concentration used here (300 μmol/L) is within the range commonly employed to induce reproducible Cd toxicity in hydroponic soybean experiments, and 200 mg/L was selected with reference to our previous studies, in which the same LW-CN concentration effectively improved thermotolerance in soybean and germination under salt stress without adverse effects on non-stressed plants [34,35]. A systematic dose–response assessment of LW-CN concentrations will nevertheless be required in future work to define the effective and safe application range. Under non-stress conditions, LW-CNs had no significant effect on soybean growth parameters, indicating that their plant-promoting effects are distinctly “stress-dependent”—that is, they do not induce redundant metabolic disturbances under normal conditions but instead exert protective roles under stress. This characteristic is consistent with the performance of LW-CNs in rapeseed [36], salt-stressed wheat [34], and heat-stressed soybean [35], and aligns with reports on Mn_3_O_4_ nanoenzymes and ZnO nanoparticles [37] alleviating Cd stress, further supporting the application positioning of plant-derived nanomaterials as “stress-regulated” biostimulants.

### 3.2. LW-CNs Maintain Carbon Assimilation by Protecting Chloroplast Structure and Photosynthetic Function

Photosynthesis is the primary target of Cd toxicity [12,32,37,38]. In this study, Cd stress caused the net photosynthetic rate (Pn) to sharply decline from 6.42 μmol·m^−2^·s^−1^ to 0.29 μmol·m^−2^·s^−1^, accompanied by a simultaneous decrease in transpiration rate (Tr) and stomatal conductance (Gs), while intercellular CO_2_ concentration (Ci) increased rather than decreased. The inverse relationship between Ci and Pn indicates that Cd-induced inhibition of photosynthesis was unlikely to arise primarily from stomatal limitation, and more probably reflected damage to mesophyll conductance and biochemical capacity [14]. Transmission electron microscopy confirmed this conclusion: under Cd stress, chloroplast morphology was distorted, thylakoid lamellae were disorganized, and plastoglobules increased—consistent with previous findings that Cd disrupts thylakoid membrane structure and PSII electron transport. After foliar application of LW-CNs, ultrastructural integrity of chloroplasts was significantly preserved, and Pn, Tr, and Gs markedly recovered, whereas Ci showed no further change. It should be noted, however, that chlorophyll fluorescence, PSII efficiency, electron transport rate, Rubisco activity, and mesophyll conductance were not directly measured in this study; therefore, the recovery of Pn can only be tentatively attributed to the restoration of non-stomatal (mesophyll/biochemical) capacity rather than to stomatal reopening, and this interpretation warrants verification with dedicated photosynthetic measurements in future work. This mechanism resembles the way foliar application of Mn_3_O_4_ nanoparticles restores rice photosynthesis by improving mesophyll conductance and biochemical capacity (Vcmax, Jmax, Rubisco activity) [14].

Chloroplasts are key subcellular sites for Cd accumulation and toxicity. Using a cadmium-responsive fluorescent probe (Leadmium™ Green AM), this study observed partial co-localization between Cd-derived fluorescence and chloroplast autofluorescence, suggesting—but not quantitatively demonstrating—that a portion of the Cd entering leaves may localize within chloroplasts and damage the photosynthetic machinery. After LW-CN treatment, the probe-derived Cd signal co-localized with chloroplasts decreased markedly (relative intensity from 0.774 to 0.171), which is consistent with the ICP-MS-based reduction in total leaf Cd and collectively supports the inference that LW-CNs partly protect the photosynthetic machinery by reducing leaf Cd accumulation. Because probe-based imaging is inherently semi-quantitative, these subcellular observations should be regarded as complementary evidence rather than independent quantification of chloroplast-specific Cd.

### 3.3. LW-CNs Systemically Enhance Antioxidant Defense and Maintain ROS Homeostasis

Cadmium can disrupt the electron transport chain via Fenton-like reactions, triggering excessive accumulation of reactive oxygen species (ROS), leading to membrane lipid peroxidation and damage to biomolecules. In this study, Cd stress significantly increased the levels of H_2_O_2_, O_2_·^−^, and MDA in leaves, while DCF/DHE fluorescent imaging clearly confirmed an oxidative burst. After foliar application of LW-CNs, all these oxidative damage indicators and fluorescence intensities significantly decreased: DCF fluorescence intensity dropped from 3.18 to 2.02, and DHE fluorescence intensity declined from 85.24 to 10.00. Mechanistically, LW-CNs markedly enhanced the activities of SOD, POD, CAT, APX, DHAR, and GR, indicating a comprehensive activation of the enzymatic defense system rather than upregulation of a single pathway. Within the AsA-GSH cycle, the central redox hub for ROS scavenging [39], DHAR and GR activities were enhanced whereas MDHAR declined [40]. Together with the decreased pools of AsA, GSH, and DHA and the marked accumulation of GSSG, these results indicate that LW-CN treatment reconfigured leaf redox metabolism toward an enzyme-driven antioxidant state: the reduced antioxidants were continuously consumed to detoxify Cd-induced peroxides, and the oxidized products consequently accumulated. Rather than a simple “acceleration” of the AsA-GSH cycle, we interpret this pattern as an altered redox balance that nevertheless sustained higher overall H_2_O_2_ clearance capacity.

The changes in non-enzymatic antioxidants presented a more complex pattern [21,41]. AsA, GSH, and DHA contents decreased, while GSSG significantly increased. This “accumulation of oxidized forms and depletion of reduced forms” pattern can be explained by two factors: (1) in response to excess ROS induced by Cd, reduced antioxidants were extensively mobilized to reduce and eliminate peroxides; and (2) GSH serves as the direct precursor for phytochelatin synthesis, and PCs chelate Cd and sequester it into vacuoles—a classic detoxification mechanism in plants [42,43,44]. Thus, the consumption of GSH may partly result from enhanced PCs synthesis. Meanwhile, osmoprotectants such as proline and soluble sugars sharply declined after LW-CN treatment (from 472.73 to 100.14 μg/g and from 81.10 to 38.10 mg/g, respectively), indicating reduced overall stress intensity and lower dependence on osmoregulation and carbon reserves, further confirming the detoxifying effect of LW-CNs. Maintaining ROS homeostasis also emerged as a core mechanism through which LW-CNs enhance soybean heat tolerance [35] and improve wheat germination under salt stress [34], highlighting the conserved cross-stress protective role of this plant-derived nanomaterial centered on redox regulation.

### 3.4. LW-CNs Mitigate Cadmium Accumulation in Plant Tissues

Restricting Cd translocation to aboveground parts and sensitive subcellular compartments represents the most direct physiological strategy to alleviate Cd toxicity [22,33,45]. In this study, LW-CNs significantly reduced Cd concentrations in roots, stems, and leaves, demonstrating that foliar application of LW-CNs systematically reduces tissue Cd accumulation and long-distance Cd transport, not merely providing localized protection. It should be noted that reduced tissue Cd concentration does not by itself establish reduced Cd uptake by roots; altered translocation, growth-related dilution, and sequestration may also contribute. Similarly, foliar application of ZnO nanoparticles has been shown to regulate root metabolism and rhizosphere microbiota via long-distance migration of Zn from leaves to roots, reducing root Cd content by 26.8–41.8% [15]. Additionally, ZnO nanoparticles can modify Cd subcellular distribution and chemical speciation by altering cell wall components such as pectin and phenolic compounds, thereby reducing Cd translocation from roots to shoots [37]. In contrast, Jiang et al. [14] reported that foliar application of Mn_3_O_4_ nanoparticles restored photosynthetic and antioxidant capacities in rice without altering Cd accumulation, suggesting divergent mitigation strategies for different nanomaterials that fall into two pathways: “Cd reduction” and “Cd tolerance.” The results of this study indicate that LW-CNs employ a dual mechanism, significantly reducing plant Cd accumulation while simultaneously enhancing antioxidant and photosynthetic protective systems. The specific mechanisms by which LW-CNs reduce tissue Cd accumulation remain to be elucidated; several possibilities—including regulation of Cd transporter gene expression (e.g., ZIP and Nramp families), enhanced cell-wall retention, direct chelation/adsorption of Cd^2+^ on nanoparticle surfaces, and basipetal translocation of LW-CNs from leaves to roots—were not directly examined in the present study and are therefore proposed strictly as working hypotheses for future investigation at the transcriptomic and nano–plant interaction levels [6,8,46,47].

### 3.5. LW-CNs Organ-Specifically Reprogram Nitrogen Metabolism

Nitrogen assimilation is another critical target of Cd toxicity [25,26,48,49]. Cd can inhibit nitrate reductase (NR), nitrite reductase (NiR), glutamine synthetase (GS), and glutamate synthase (GOGAT), forcing ammonia assimilation to shift from the primary GS/GOGAT pathway toward the GDH bypass, accompanied by tissue ammonium accumulation and protein degradation [50,51]. This study reveals that LW-CNs regulate nitrogen metabolism under Cd stress in an organ-specific manner. In roots, LW-CNs significantly upregulated NR activity (by 95.9%) and concurrently increased GOGAT (+61.3%) and GDH (+68.4%) activities, leading to substantial accumulation of glutamate, while total nitrogen and ammonium nitrogen remained stable. These data indicate enhanced capacity of root nitrate reduction and ammonium assimilation, supporting a pattern of “root-zone nitrogen assimilation and storage.” This finding aligns with the adaptive strategy proposed by Chaffei et al. [51]—“nitrogen remobilization in leaves and nitrogen storage in roots under Cd stress”—and extends the previously observed effects of LW-CNs in promoting nitrogen uptake and assimilation under heat stress in soybean [35] and salt stress in wheat [34].

In leaves, LW-CNs significantly enhanced NR activity but markedly reduced GS, GOGAT, GDH, GOT, and GPT activities, along with Glu and Gln contents, while ammonium nitrogen accumulated and total nitrogen decreased. Notably, these changes reflect altered enzyme-activity patterns rather than enhanced nitrogen assimilation in leaves; in particular, the concurrent ammonium accumulation and decreased GS/GOGAT activity may indicate partial suppression of leaf ammonium assimilation. Combined with natural-abundance ^15^N data showing a slight decrease in root ^15^N abundance and small but statistically significant increases in stem and leaf ^15^N abundance, these results are consistent with an altered distribution of nitrogen among organs. Because the isotope shifts were small and no exogenous ^15^N tracer was applied, this evidence supports—but does not directly demonstrate—enhanced nitrogen translocation from roots to aboveground parts; a dedicated ^15^N-labeling tracer experiment will be required to quantify the actual flux (see Section 4.9). The decline in leaf total nitrogen and nitrogen metabolite concentrations may be associated with growth recovery-induced “dilution effect” and redistribution of nitrogen metabolic fluxes. The uncoupling between NR and GS/GOGAT activities in the leaves suggests that while nitrate reduction capacity increases, ammonia assimilation and recycling are downregulated. The physiological significance of this phenomenon—potentially serving as a signaling node for nitrogen redistribution or interacting with photorespiratory ammonia release—warrants further investigation through transcriptomics and isotope tracing [52,53,54]. Overall, LW-CNs do not simply promote nitrogen metabolism uniformly but instead precisely reprogram it according to organ function. Together with their central role in maintaining ROS homeostasis, these effects form the physiological basis for mitigating Cd toxicity.

## 4. Materials and Methods

### 4.1. Plant Material, Growth Conditions, and Experimental Design

The widely cultivated soybean variety Xin Da Dou 26 was used as the experimental material. Seeds were surface-sterilized with a 1% sodium hypochlorite solution and then thoroughly rinsed with sterile deionized water. Sterilized seeds were sown in nutrient pots for germination. After cotyledons fully expanded, uniform seedlings were selected and transplanted into black hydroponic containers (16.5 cm × 12.5 cm × 5 cm) filled with 1 L of 1/2-strength Hoagland nutrient solution (pH 6.0 ± 0.2), with six plants per container; three independent containers were used per treatment. The nutrient solution was renewed every 3 days throughout the experiment. Plants were grown under controlled environmental conditions: day/night temperatures of 28 ± 1 °C/25 ± 1 °C, relative humidity of 70%, photoperiod of 14/10 h, and photosynthetic photon flux density of 200 μmol m^−2^ s^−1^.

Four treatments were set up: (1) non-stress control; (2) application of LW-CNs without cadmium stress; (3) cadmium stress control; (4) application of LW-CNs under cadmium stress. LW-CNs were applied foliarly at 200 mg/L; 3 h later, cadmium stress was initiated by adding CdCl_2_ to the nutrient solution at a final concentration of 300 μmol/L. The Cd-containing nutrient solution was renewed every 3 days with freshly prepared solution of the same Cd concentration, so that the Cd stress was maintained constant throughout the 7-day experimental period. For foliar application, the LW-CN suspension was sprayed onto the seedlings with a hand-held atomizer at approximately 0.05 mL per plant, applied evenly onto both the adaxial and abaxial leaf surfaces until the foliage was uniformly wetted; Silwet L-77 as a surfactant was added. During spraying, the hydroponic containers were covered with plastic film to prevent contamination of the nutrient solution, and the treated plants were allowed to air-dry before being returned to the growth chamber.

### 4.2. Preparation and Characterization of LW-CNs

Licorice–Lycium barbarum-based nanocomposites (LW-CNs) were prepared via a hydrothermal method. The synthesis protocol was identical to that used in our previous studies, in which the physicochemical properties of the material obtained by this protocol were characterized [28,31,32]; these characterizations were therefore not repeated in the present study. It should be acknowledged that a control group treated with a licorice–wolfberry extract prepared without hydrothermal processing was not included. Licorice and Lycium barbarum were separately ground into powders; 200 mg of each powder was mixed thoroughly and dispersed in 20 mL of ultrapure water. The resulting suspension was transferred into a stainless steel autoclave lined with polytetrafluoroethylene (PTFE), sealed, and reacted at 160 °C for 6 h. After cooling to room temperature, the reaction mixture was aliquoted into 2 mL centrifuge tubes and centrifuged at 4000 rpm for 30 min. The red-brown supernatant was collected as LW-CNs and stored at 4 °C for the subsequent experiments [28].

### 4.3. Growth Phenotype and Biomass Measurement

Whole-plant phenotype images were taken on the seventh day after treatment. Plant height was measured from the cotyledon node to the apical meristem. Aboveground parts and roots were separated, surface moisture gently removed with absorbent material, and immediately weighed to obtain fresh aboveground biomass and fresh root biomass. Six biological replicates were performed for each treatment.

### 4.4. Observation of Chloroplast Ultrastructure

Leaf tissue blocks were excised and immediately fixed in 2.5% glutaraldehyde solution, followed by postfixation in 1% osmium tetroxide at room temperature in the dark for 2 h. Tissues were then dehydrated through a graded ethanol series and embedded in Spurr low-viscosity resin. Ultrathin sections were stained with uranyl acetate and lead citrate, and observed using a transmission electron microscope (JEM-1400, JEOL, Akishima, Japan) at an accelerating voltage of 120 kV.

### 4.5. Measurement of Leaf Gas Exchange Parameters

Leaf net photosynthetic rate (Pn), transpiration rate (Tr), stomatal conductance (Gs), and intercellular CO_2_ concentration (Ci) were measured using a portable photosynthesis system (LI-6800, LI-COR Biosciences, Lincoln, NE, USA). Measurements were conducted one hour after lights-on in the morning, with a leaf chamber light intensity of 1200 μmol m^−2^ s^−1^, reference CO_2_ concentration of 400 μmol mol^−1^, leaf chamber temperature of 25 °C, and relative humidity of approximately 60%. Data were recorded once the readings stabilized.

### 4.6. Determination of Oxidative Damage, Osmoprotectants, and Antioxidant Metabolism

Fresh leaves from the same leaf position of each treatment group were rapidly frozen in liquid nitrogen and stored at −80 °C. Hydrogen peroxide (H_2_O_2_) content and superoxide anion (O_2_·^−^) production rate were determined using the potassium iodide method and hydroxylamine oxidation method, respectively. Malondialdehyde (MDA) content was measured using the thiobarbituric acid method. Soluble sugar and proline contents were determined using the anthrone colorimetric method and ninhydrin acid method, respectively. SOD activity was determined using the nitroblue tetrazolium photoreduction inhibition method, while POD, CAT, and APX activities were measured by monitoring changes in absorbance at 470, 240, and 290 nm, respectively. MDHAR, DHAR, and GR activities were assessed based on NADH oxidation, AsA production, and NADPH oxidation rates, respectively. AsA and total ascorbate (AsA + DHA) contents were determined using the bipyridyl colorimetric method, with DHA content calculated as the difference between total ascorbate and AsA. Total glutathione and GSSG contents were measured using the DTNB enzyme cycling method, and GSH content was calculated by subtracting twice the GSSG content from the total glutathione content. The AsA/DHA and GSH/GSSG ratios were further calculated to evaluate leaf redox homeostasis. All measurements included three independent biological replicates.

### 4.7. Intracellular Reactive Oxygen Species Fluorescence Imaging

Reactive oxygen species in leaf cells were visualized using 2′,7′-dichlorodihydrofluorescein diacetate (H_2_DCFDA) and dihydroethidium (DHE) under a laser confocal microscope to assess overall oxidative activity and superoxide anion-related fluorescence. Leaf samples were incubated in the dark for 30 min, washed three times, and then imaged. Imaging parameters were set as follows: excitation at 488 nm; PMT1, 500–600 nm (for detecting DCF and DHE fluorescence) and 500–580 nm (for HPF fluorescence); PMT2, 700–785 nm (for chloroplast autofluorescence).

### 4.8. Cadmium Tissue Localization and Quantification

Cadmium ions in plant tissues were imaged using a cadmium-responsive fluorescent probe under a laser confocal microscope, with excitation at approximately 514 nm, and chloroplast autofluorescence collected in the 663–738 nm range. Samples were incubated in a working solution of the probe (Leadmium™ Green AM) at a final concentration of 10 μmol/L (prepared in sterile 0.85% saline, used immediately). For the determination of total tissue cadmium content, harvested samples were successively rinsed with 20 mmol/L EDTA-Na_2_ desorption solution and deionized water, then blanched at 105 °C for 30 min, dried at 75 °C to constant weight, and digested wetly with a mixture of nitric acid and perchloric acid. After overnight cold digestion, samples were gradually heated on a hot plate until the solution became clear and nearly dry. The solution was cooled, diluted to 25 mL with ultrapure water, and analyzed for cadmium content using inductively coupled plasma mass spectrometry (ICP-MS, Agilent 7900, Agilent Technologies, Santa Clara, CA, USA). Certified reference materials, procedural blanks, and calibration verification samples were included during analysis. Final cadmium concentrations were expressed and calculated relative to plant dry weight.

### 4.9. Nitrogen Status and Nitrogen Assimilation Metabolism

Total nitrogen content in roots and leaves was determined by elemental analysis. Fresh root and leaf samples were blanched at 105 °C for 30 min, dried at 75 °C to constant weight, ground, and passed through a 100-mesh sieve. Five to eight milligrams of dry sample were sealed in tin capsules and analyzed for total nitrogen using an elemental analyzer, with nitrogen content normalized to dry weight. Nitrate reductase (NR) and nitrite reductase (NiR) activities were determined using the sulfanilamide–naphthylethylenediamine colorimetric method, normalized to nitrite production per gram fresh weight per hour.

Nitrogen uptake dynamics in soybean were investigated using ^15^N-labeled urea, which was applied to the root system with a final ^15^N abundance of 0.5%. The abundance of ^15^N in roots, stems, and leaves was measured using an isotope ratio mass spectrometer (IRMS; Delta V Advantage, Thermo Fisher Scientific, Bremen, Germany) coupled to an elemental analyzer. Dried, ground samples (2–3 mg) were sealed in tin capsules and combusted at 1020 °C, and the produced N_2_ was analyzed for its ^15^N/^14^N isotope ratio. No exogenous ^15^N-labeled fertilizer was applied in this experiment; the reported values therefore represent natural-abundance ^15^N, expressed as atom % ^15^N and calculated as atom % ^15^N = 100 × ^15^N/(^15^N + ^14^N).

Glutamine synthetase (GS), glutamate oxoglutarate aminotransferase (GOGAT), glutamate dehydrogenase (GDH), aspartate aminotransferase (GOT), and alanine aminotransferase (GPT) activities, as well as ammonium nitrogen, glutamate, glutamine, and crude protein contents were determined using reagent kits from Pnuis Company, Hong Kong, China. All enzyme extracts were prepared at 4 °C. Enzyme activity was expressed as the amount of product generated per gram of fresh weight per hour. Roots and leaves were measured independently, with three biological replicates for each parameter.

### 4.10. Statistical Analysis

Experimental data were organized and calculated using Microsoft Excel 2023. Data were analyzed by two-way analysis of variance (ANOVA) with Cd stress and LW-CN application as fixed factors using SPSS 23 software (SPSS Inc., Chicago, IL, USA). Where the ANOVA indicated a significant effect, differences among the four treatment groups were further compared using Tukey’s honestly significant difference (HSD) test, which controls the family-wise Type I error rate. Growth parameters were analyzed with six biological replicates (*n* = 6), and all physiological and biochemical parameters with three biological replicates (*n* = 3). Three independent containers were maintained per treatment; each biological replicate of the biochemical analyses originated from an independent container, and technical replicates were measured to verify analytical reproducibility. Exact *p* values for all statistical comparisons are provided in Supplementary Tables S1 and S2. Graphs were created using GraphPad Prism 10 software (GraphPad Software Inc., San Diego, CA, USA). ns, *p* ≥ 0.05; *, *p* < 0.05; **, *p* < 0.01; ***, *p* < 0.001; ****, *p* < 0.0001.

## 5. Conclusions

Cadmium (Cd) pollution poses substantial risks to soybean growth by impairing photosynthetic performance, triggering excessive ROS buildup, disrupting nitrogen metabolism, and causing severe cellular damage. Plant-derived nanomaterials hold great promise for mitigating abiotic stress in crops, yet few studies have provided thorough physiological documentation of how LW-CNs defend soybean against Cd-induced phytotoxicity. In the present work, LW-CNs produced by one-step hydrothermal synthesis were applied as foliar sprays to Cd-challenged soybean seedlings, aiming to unpack their underlying protective mechanisms. Combined phenotypic, physiological and subcellular observations show that LW-CNs relieve Cd toxicity via four coordinated physiological pathways. Firstly, LW-CNs maintain intact chloroplast ultrastructure and recover net photosynthetic capacity apparently through largely non-stomatal routes, offsetting Cd-driven harm to the photosynthetic apparatus. Secondly, LW-CNs trigger a comprehensive antioxidant defense response, elevating the activities of SOD, POD, CAT, APX, DHAR and GR, while reconfiguring the redox status of the AsA–GSH cycle. Such changes promote effective ROS clearance, limit lipid peroxidation, and reinstate cellular redox homeostasis under Cd stress. Thirdly, foliar LW-CN treatment lowers Cd levels across roots, stems and leaves, reduces Cd co-localization inside chloroplasts, and lessens direct subcellular damage to photosynthetic machinery. Fourthly, LW-CNs bring about organ-specific reshaping of nitrogen metabolism: root-localized nitrate reduction and ammonium assimilation are strengthened, the redistribution of nitrogen from the roots to aboveground parts is enhanced, and whole-plant nitrogen partitioning is re-adjusted under Cd exposure. Notably, LW-CNs confer stress-targeted protection and produce little harmful effect on soybean growth under non-stressed conditions, supporting their potential as plant-based agricultural biostimulants, whose residual fate, persistence, and effects on non-target organisms should be further evaluated before large-scale agricultural deployment. This work presents systematic physiological data illustrating the multifaceted action of LW-CNs for soybean Cd tolerance, covering Cd-accumulation suppression, photosystem safeguarding, redox homeostasis and organ-dependent nitrogen redistribution. Our results deepen the mechanistic knowledge of plant-derived nanomaterial-governed heavy metal resistance and furnish experimental references for developing nano-biotech solutions to sustain soybean production on Cd-polluted farmland.

## Data Availability

The data presented in this study are available on request from the corresponding author.

## Supplementary Materials

The following supporting information can be downloaded at https://www.mdpi.com/article/10.3390/plants15213282/s1, Table S1: Exact *p* values of the two-way ANOVA; Table S2: Exact *p* values of independent samples t test.
