# Supplementary material for: Foliar-Applied LW-CNs Alleviate Cadmium Toxicity in Soybean by Regulating Cd Accumulation, Photosynthesis and Nitrogen Metabolism
Source: Plants (Basel). 2026 Oct 24;15(21):3282. doi: 10.3390/plants15213282 (PMC13765685; doi:10.3390/plants15213282)
Supplement: Supplementary file 1 [file plants-15-03282-s001.zip › plants-4640359-supplementary.pdf]

Table S1. Exact P values of the two-way ANOVA.

Parameter (Section)	P (Cd)	P (LW-CNs)	P (Cd × LW-CNs)	P (CK vs LW-CNs)	P (CK vs Cd)	P (Cd vs Cd + LW-CNs)	P (LW-CNs vs Cd + LW-CNs)
**2.1 Growth**							
Plant height	0.0001	0.355	0.195	0.706	＜0.0001	0.24	0.002
Shoot fresh weight	0.0002	0.0006	0.0006	1	＜0.0001	＜0.0001	0.985
Root fresh weight	＜ 0.0001	0.0002	0.0035	0.824	＜0.0001	＜0.0001	0.0395
**2.2 Gas exchange**							
Photosynthetic rate（Pn)	＜ 0.0001	0.0003	0.0008	0.0112	＜0.0001	0.0276	＜0.0001
Transpiration rate (Tr)	＜ 0.0001	＜0.0001	＜0.0001	＜0.0001	＜0.0001	0.0557	＜0.0001
Stomatal conductance (Gs)	＜ 0.0001	＜0.0001	0.0887	＜0.0001	＜0.0001	0.0105	＜0.0001
Intercellular CO_2 concentration (Ci)	＜ 0.0001	0.2611	0.1793	0.3016	＜0.0001	0.9983	＜0.0001

Table S2. Exact P values of independent-samples t-test.

Parameter (Section)	P (Cd vs Cd + LW-CNs)
**2.3 Oxidative damage**	
H_2O_2 content	0.0013
$O_2^{\cdot -}$ production rate	0.0011
MDA content	＜0.0001
Soluble sugar content	＜0.0001
**2.3 Antioxidant enzymes**	
SOD activity	＜0.0001
POD activity	＜0.0001
CAT activity	0.0007
APX activity	0.0001
MDHAR activity	0.0019
DHAR activity	＜0.0001
GR activity	＜0.0001
**2.3 AsA–GSH pool**	
ASA content	＜0.0001
GSH content	0.0001
GSSG content	＜0.0001
DHA content	＜0.0001
GR content	＜0.0001
PRO content	＜0.0001
**2.3 In-situ ROS imaging**	
DCF fluorescence intensity	0.0064
DHE fluorescence intensity ($O_2^{\cdot -}$)	＜0.0001
**3.5 N metabolism**	
NiR activity (root）	0.3461
NiR activity（leaf）	0.0007
GS activity (root)	0.5719
GS activity (leaf)	0.0003
GOGAT activity (root）	＜0.0001
GOGAT activity（leaf）	＜0.0001
GDH activity (root）	＜0.0001
GDH activity（leaf）	＜0.0001
GOT activity (root）	0.362
GOT activity（leaf）	0.0118
GPT activity (root）	＜0.0001
GPT activity（leaf）	＜0.0001
Glutamate (Glu) content (root）	＜0.0001
Glutamate (Glu) content（leaf）	＜0.0001
Glutamine (Gln) content（leaf）	＜0.0001
Ammonium nitrogen content (root）	0.1458
Ammonium nitrogen content（leaf）	＜0.0001
Total nitrogen content (root）	0.2257
Total nitrogen content（leaf）	0.0028
**3.5 Isotope**	
Atom % ^{15}N (stem)	＜0.0001
Atom % ^{15}N (leaf)	0.0493
**2.4 Cd accumulation**	
Cd content (stem)	＜0.0001
Cd content (leaf)	＜0.0001
